# The Comparative Toxicity of 10 Microcystin Congeners Administered Orally to Mice: Clinical Effects and Organ Toxicity

**DOI:** 10.3390/toxins12060403

**Published:** 2020-06-18

**Authors:** Neil Chernoff, Donna Hill, Johnsie Lang, Judy Schmid, Thao Le, Amy Farthing, Hwa Huang

**Affiliations:** 1 Center for Public Health & Environmental Assessment, U.S. Environmental Protection Agency, Research Triangle Park, NC 27711, USA; Hill.Donna@epa.gov (D.H.); jeschmid@mebtel.net (J.S.); 2 Oak Ridge Institute for Science and Education, Oak Ridge, TN 37830, USA; jrlang@ncsu.edu (J.L.); thaole77@gmail.com (T.L.); alokichi@ncsu.edu (A.F.); hira719314@gmail.com (H.H.)

**Keywords:** HAB, cyanobacteria, cyanotoxin, microcystin, hepatic toxicology, oral administration, mouse

## Abstract

Microcystins (MCs) are common cyanobacterial toxins that occur in freshwaters worldwide. Only two of the >200 MC variants have been tested for potential toxicity after oral exposure. This paper reports on the toxicity of 10 different MC congeners identified in algal blooms, microcystin-LR (MCLR), MCLA, MCLF, MCLW, MCLY, MCRR, [Asp3]MCRR, [Asp3,Dhb7]MCRR, MCWR, and MCYR after single administrations to BALB/c mice. In a preliminary MCLR dose–response study of 3 to 9 mg/kg doses, ≥5 mg/kg induced clinical changes, increased serum levels of ALT, AST, and GLDH, liver congestion, increased liver/body weight ratios, and reduced serum glucose and total protein. Based on the extent of these effects, the 10 congeners were administered as single 7 mg/kg oral doses and toxicity evaluated. The greatest toxicity was observed with MCLA and MCLR including a high percentage of moribundity. In addition to eliciting effects similar to those listed above for MCLR, MCLA also induced serum alterations indicative of jaundice. MCLY, and MCYR induced changes like those noted with MCLR, but to lesser extents. MCLW and MCLF exhibited some serum and morphological changes associated with hepatic toxicity, while there were few indications of toxicity after exposures to MCRR, [Asp^3^]MCRR, [Asp^3^,Dhb^7^]MCRR, or MCWR. These data illustrate a wide spectrum of hepatic effects and different potencies of these MC congeners.

## 1. Introduction

Cyanobacteria constitute a phylum of photosynthetic gram-negative bacteria that are among the most common freshwater organisms on the planet. Cyanobacteria are present in all freshwater systems and are known to undergo episodic periods of rapid increase of biomass that may be accompanied by high levels of toxins (harmful algal blooms (HABs)) that induce adverse health effects in vertebrates including humans [1,2,3,4]. Blooms may be caused by a combination of factors, including nitrogen and phosphorus levels [5,6,7], water column stability [8], and increased temperatures [9,10]. The production of toxins by HABs has been correlated with increased eutrophication [11,12] and water temperatures [13]. Adverse cyanotoxin-induced health effects in vertebrates include neurotoxicity from anatoxins and saxitoxins [14,15] and hepatotoxicity from cylindrospermopsin, microcystins (MCs), and nodularin [16,17,18,19]. Surveys of potential associations of cyanotoxin exposures through HABs in United States (U.S.) freshwaters found that MCs were the most common cyanotoxins present [20,21]. 

MCs are associated with illness and deaths in wildlife [22,23]; cattle [24,25]; and dogs [26,27]. Environmental exposures to freshwater MCs may also occur in marine environments [28] and have been implicated in the deaths of sea otters [29]. MCs caused severe toxicity in humans after exposure in a dialysis clinic [30,31]. Possible links of MC recreational and/or drinking water exposures and human illness have been reviewed [32], and while there are numerous suggestions of links between primary liver cancer and environmental MC exposures, there are currently no definitive conclusions about this relationship. There are indications that low levels of MC exposures in children through food and drinking water may be associated with liver damage as determined by serum markers for hepatotoxicity [33], and exposure through drinking water has been associated with gastrointestinal symptoms [34]. MC exposure through consumption of freshwater fish is also another possible route of human exposure [35].

MCs are among the most common groups of cyanobacterial toxins. They are cyclic heptapeptides with five non-proteinogenic amino acids including 3-amino-9-methoxy-2,6,8-trimethyl-10-phenyl-4,6-decadienoic acid (ADDA), that has been found only on these compounds, and the structurally related cyclic pentapeptide nodularins [36]. Common variations of MCs involve substitution of the two proteinogenic amino acids in the second and fourth positions of the peptide. MCLR, for example, contains leucine (L) and arginine (R). Many additional MC variants have been identified based on changes in the other amino acids [19,37,38,39,40]. The major cyanobacterial genus producing MCs in the United States is *Microcystis* but MCs are also known to be produced by species in other common genera including *Dolichospermum* (ex *Anabaena), Oscillatoria, Nostoc,* and *Planktothrix* [41,42,43]. Toxins found in midwestern U.S. lake HABs were predominantly either MCRR or MCLR, but some contained high levels of other MCs including MCLA and MCYR, which were also the predominant MCs in several lakes [44]. Most of the MC toxicology studies have involved MCLR [45] because of its common occurrence and potency [46]. 

Studies using the intraperitoneal (i.p.) route of administration in mice demonstrate that MCLR is primarily a hepatic toxin. Once in the blood, MCLR reaches the liver where it is transported through the hepatocyte cell membranes by organic anion transporting polypeptides (OATPs), most notably Oatp1b2 in the mouse and the human orthologs OATP1B1 and OATP1B3 [47,48,49,50]. In the hepatocyte, MCLR induces cellular toxicity by several mechanisms including formation of reactive oxygen species with resultant oxidative stress, DNA damage and apoptosis [51,52], resulting in liver toxicity and dysfunction. Another major mechanism of toxicity involves inhibition of protein phosphatases 1 (PP1) and 2A (PP2A), resulting in hyperphosphorylation that disrupts the cytoskeleton and causes cellular breakdown and death [53,54,55]. Acute toxicity resulting from high levels of MCs produces massive bleeding in the liver, necrosis, and hemorrhagic shock [56,57]. 

There is a series of studies in which mice were exposed to MCLR by the oral route of administration [4]. Acute exposure to MCLR resulted in an estimated 50% lethal dose (LD50) of 10.9 mg/kg [58]. Significant elevations in serum alanine amino transferase (ALT) and aspartate amino transferase (AST) occurred after dosing animals by gavage with ≥200 µg/kg MCLR for three months, and at the 1000 µg/kg dose, reduced albumin and total protein, inflammation and focal degeneration of hepatocytes were observed [59]. MCLR administered daily to rats at 50 or 100 µg/kg/day orally for one month induced dose-related increases in liver weight, alkaline phosphatase (ALP), degenerative hepatocytes, and hemorrhages [60]. Oral exposures of MCLR to 50 or 100 µg/kg administered to mice on alternate days for one month were investigated and both dose levels induced increased cytoplasmic vacuolation and steatosis in the centrilobular zone and reduced superoxide dismutase activity in treated hepatocytes [61]. MCRR administered by the oral route for seven consecutive days at doses of 4.6–186 µg/kg found that, at levels of ≥46 µg/kg/day, the terminal deoxynucleotidyl transferase dUTP Nick-End Labeling (TUNEL) assay indicated significant incidences of apoptosis in hepatocytes [62]. There do not appear to be oral route studies with the other MC congeners. 

This paper reports the toxicity induced in BALB/c mice after single oral doses of 10 MC congeners (MCLA, MCLF, MCLR, MCLW, MCLY, MCRR, [Asp3]MCRR, [Asp3,Dhb7]MCRR, MCWR, and MCYR) that include eight not previously studied by this route of administration. These were chosen because they have been identified in U.S. waters [44] and globally [37]. We established the comparative toxicity of these congeners using endpoints assessing hepatic function and homeostasis. Initially, MCLR was evaluated with a range of dose levels from 3 to 9 mg/kg to establish a dose that would be used to compare toxicity of the congeners. The selected dose, 7 mg/kg, was chosen because it is a significantly toxic dose of MCLR that allows for a considerable range of variation when evaluating the comparative toxicity of the other congeners. These studies are an initial effort to evaluate the comparative toxicities of a wide array of microcystins found in the environment using the appropriate oral route of administration in laboratory animals. 

## 2. Results

### 2.1. MCLR Dose Response After Oral Administration 

**Figure 1:**
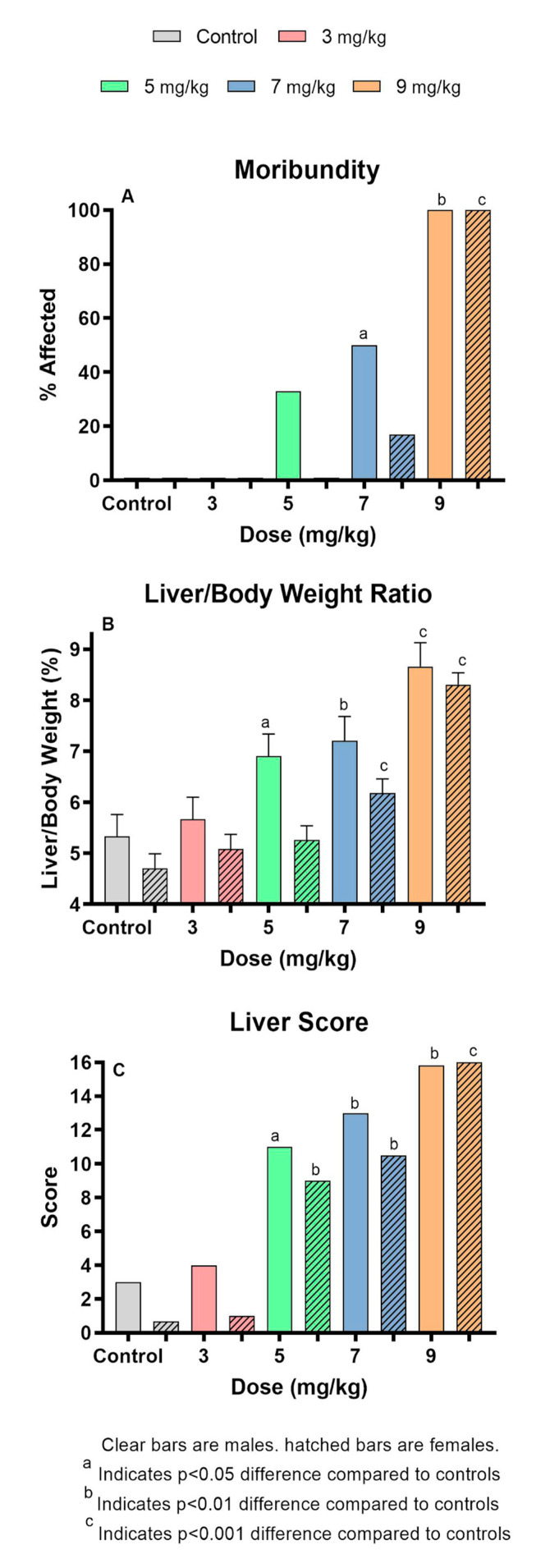
Effect of single administration of different levels of MCLR to the Balb/c mouse. (**A**). Extent of induced moribundity, a measure of severe toxicity; (**B**). Liver/body weight ratios that indicate increased relative weight and/or size of livers; (**C**). Liver score, a general measure of gross hepatic appearance at the time of necropsy.

The MCLR dose–response data that were used to select the 7 mg/kg dose used in this study are shown in Table 1. Weight losses were recorded in all dose groups and controls, probably reflecting the stress of the novel metabolism cage environment. No significant dose-related effects were found for any endpoints at 3 mg/kg. At ≥5 mg/kg, animals of both sexes exhibited multiple toxic effects. Effects generally included weight loss and moribundity. The frequency of moribundity increased with dose and reached 100% at 9 mg/kg (Figure 1A). Animals exposed to 9 mg/kg were euthanized an average of 6h post-dosing, as compared to 24h post-dosing for other dose groups. 

The hepatic system was significantly affected at ≥5 mg/kg, with increased liver scores and liver weights associated with congestion from intrahepatic hemorrhage, as reflected in elevated liver/body weight (L/BW) ratios (Figure 1B). Although there were increased liver scores (Figure 1C) and liver/body weight ratios in both sexes; compared to males, females did not show significant effects in the L/BW below 7 mg/kg whereas it was significantly elevated in the 5 mg/kg males. Significant increases in serum markers of liver toxicity (ALT and AST) occurred in both sexes at doses ≥5 mg/kg, and glutamate dehydrogenase (GLDH) was elevated in females at 5 and 7 mg/kg. Other significant effects became apparent at higher dose levels. Females had significantly higher BUN/creatinine at doses ≥7 mg/kg. Total protein levels were reduced in males and females at 9 mg/kg. Males at 7 mg/kg had significantly decreased glucose (*p* ≤ 0.05) and no significant changes were seen with levels of total bilirubin in either sex.

The dose response study indicated that ≥5 mg/kg induced significant toxicity with incidences of moribundity, changes in body and liver weights, and serum markers indicative of hepatic toxicity and general metabolic homeostasis. Higher dose levels induced greater effects in all parameters. The NOAEL was established as the 3 mg/kg dose level for MCLR. The 7 mg/kg dose level was selected for the comparative congener studies because it induced a degree of toxicity that could be compared to MCs that had greater or lesser toxicity.

### 2.2. Comparative Toxicity of Microcystin Congeners MCLA, MCLF, MCLR, MCLW, MCLY, MCRR, [Asp3]MCRR, [Asp3,Dhb7]MCRR, MCWR, and MCYR Administered a Single Oral Dose of 7mg/kg

**Figure 2:**
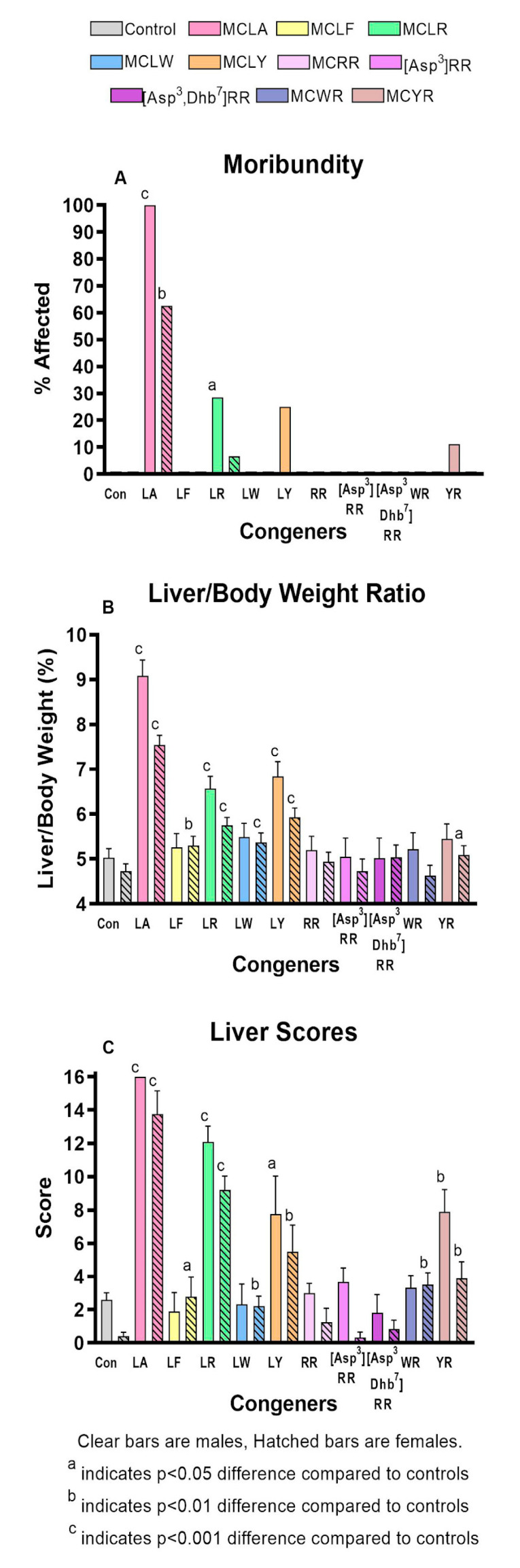
MC-induced moribundity and changes in liver weight and appearance for the 10 MC congeners studied. (**A**). Moribundity percentage (**B**). Liver/body weight ratios (**C**). Liver scores.

The comparative toxicity data for the MC congeners are summarized in supplementary Table S1. As in the MCLR dose-finding study, all groups including controls lost weight after dosing. Significantly greater weight losses occurred in both sexes of the MCLR group, in female MCLA, MCLF, and MCLY groups and in male MCYR animals. Significant incidences of moribundity (Figure 2a) were induced with MCLA (100% in males and 67% in females) and MCLR (33% in males). Non-significant rates of moribundity were also observed in MCLR females and MCLY and MCYR males. In all congeners where moribundity was induced, effects in males were greater than in females.

**Figure 3:**
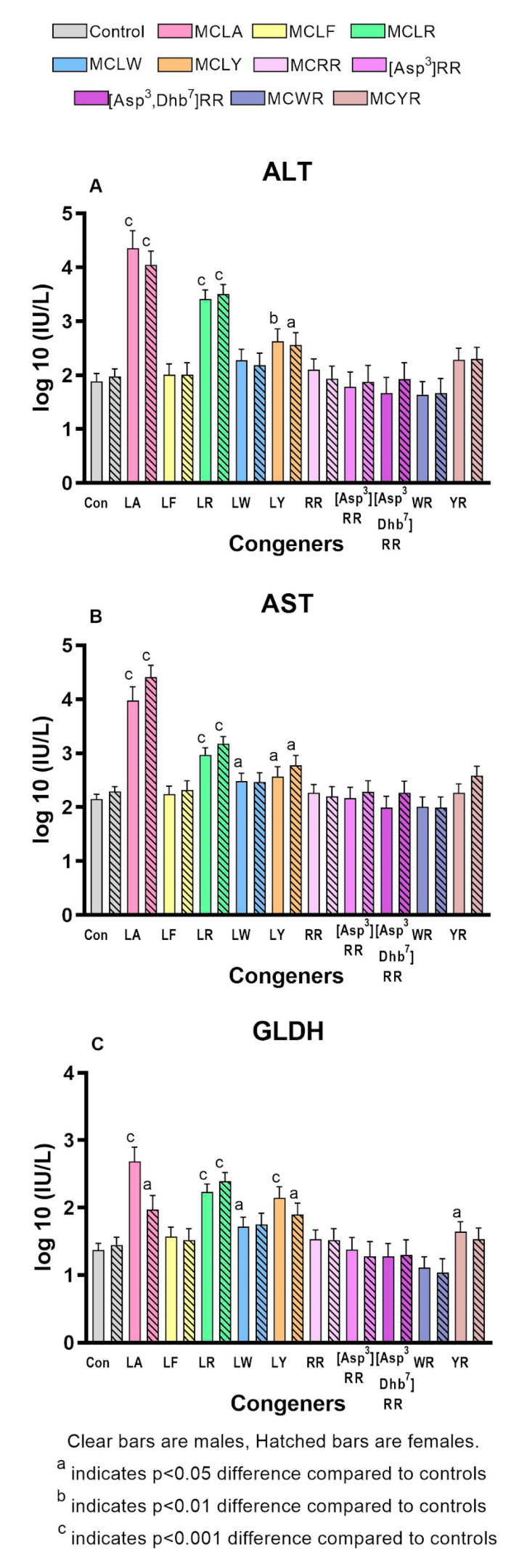
Serum markers indicative of hepatic damage and/or dysfunction. (**A**). Alanine amino transferase (ALT) (**B**). Aspartate amino transferase (AST) (**C**). Glutamate dehydrogenase (GLDH).

**Figure 4:**
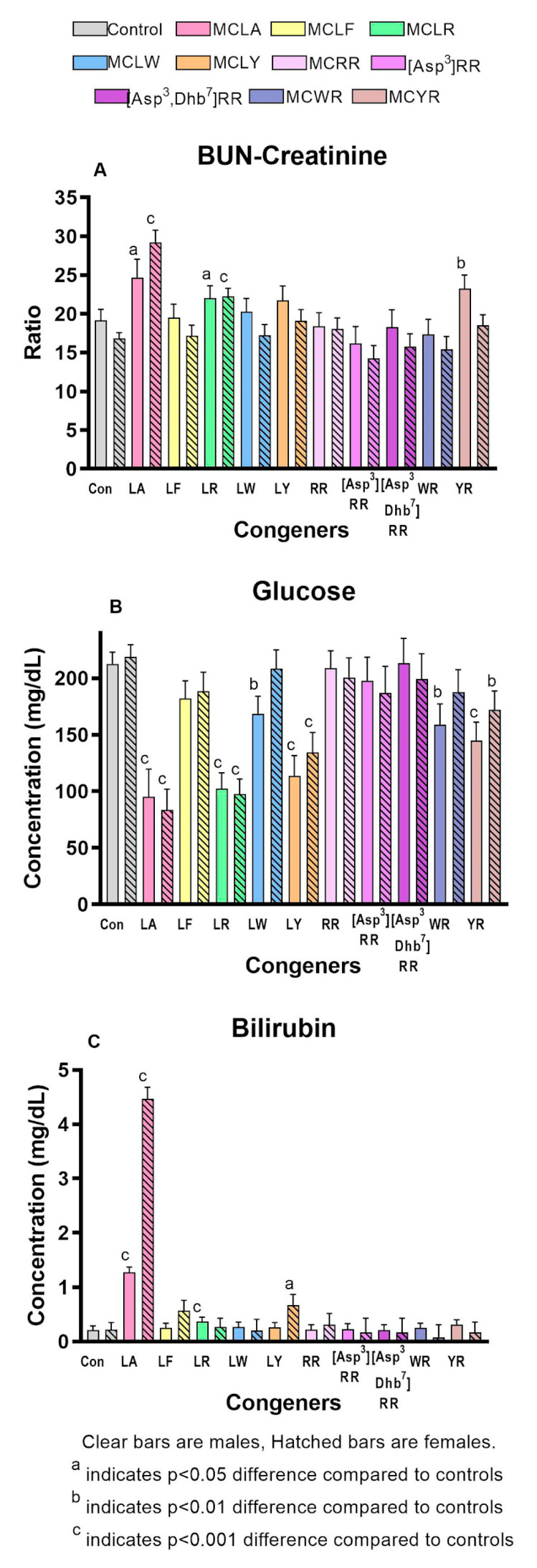
Serum measures of general homeostasis. (**A**). Blood-Urea Nitrogen / creatinine (BUN/creatinine) ratio (**B**). Serum glucose levels (**C**). Serum total bilirubin.

Significantly increased liver scores were seen in both sexes of the MCLA, MCLR, and MCLY groups and in the MCYR males (Figure 2b). The liver weights and L/BW ratios (Figure 2c) were significantly increased in both sexes of the MCLA, MCLR and MCLY groups, and only in females exposed to MCLF, MCLW. L/BW increase was the sole effect noted in animals exposed to MCYR. Significant increases in serum ALT, AST, and GLDH (Figure 3a–c) were seen in MCLA, MCLR, and MCLY groups and in the male MCLW animals. Albumin, globulin, and total protein levels were significantly reduced in the MCLA males. BUN/creatinine levels were elevated significantly in MCLA, MCLR animals of both sexes, and in male MCYR animals (Figure 4a). Significantly lower glucose levels were found in MCLA, MCLR, MCLY, and MCYR animals in both sexes, and in male MCLW and MCWR groups (Figure 4b). The serum of the MCLA group had a yellowish color unlike any other treated groups and highly significantly elevated bilirubin levels of 1.44 and 4.29 mg/dL in males and females, respectively, as compared to 0.22 and 0.24m g/dL in the corresponding controls. Bilirubin levels were also significantly elevated in the MCLR and MCYR males but to a lesser extent (0.38 and 0.33mg/dL, respectively) than MCLA animals (*p* < 0.001) (Figure 4c). The 7 mg/kg dose of MCLA induces significantly higher levels of toxicity when compared to MCLR. The endpoints showing this difference include moribundity, liver wt., L/BW ratio, liver score, elevated AST in both sexes; and elevated ALT, GLDH, BUN, albumin, globulin, and total protein in males and reduced creatinine and BUN/creatinine ratio in females (Table 2).

## 3. Discussion

MCLR dose–response data demonstrated characteristic MC hepatic toxicity after 5 mg/kg was administered as a single oral dose. Elevated dose-related incidences of moribundity, increased liver weights and liver/body weight ratios, and liver scores indicated general hepatic effects. Hepatic toxicity was demonstrated by increased levels of serum markers for hepatic injury and dysfunction (ALT, AST, GLDH). Systemic toxicity was evident with decreased total protein and serum glucose levels seen at the higher dose levels. Dose-related weight losses probably reflected systemic toxicity that resulted in reduced food and water intake at 5 and 7 mg/kg over a 24hr period. The effects noted after both 7 and 9 mg/kg doses are consistent with those observed in environmental poisonings that have occurred in both dogs [23] and cattle [24]. Significantly smaller changes in body weight and blood glucose in the 9 mg/kg groups are probably associated with reduced time to euthanasia and, therefore, less time spent in the metabolism cages prior to euthanasia. Increased serum BUN also occurred at ≥5 mg/kg and is known to be associated with a wide variety of conditions including non-alcoholic fatty acid liver disease (NAFLD) [63], renal dysfunction, and general inflammation. NAFLD has been identified in the livers of mice treated with long-term oral exposures to MCLR [64], but in this study this effect may have been confounded by stress-related decreased water intake leading to dehydration since control groups also had reduced weight gain during their residence in the metabolism cages. 

Toxicity of MCLR administered by the oral route was significantly less than after i.p. administration on a mg/kg basis. The MD50 (50% Moribundity Dose) of MCLR in this study was 7.8 mg/kg compared to LD50′s of 36 to 65 µg/kg with the i.p. route [46,58] and supports the conclusion that i.p. administration of MCLR is 100–200X more toxic than oral exposure. Similar differences between oral and i.p. administration of MCLR were reported using the comet assay that showed no DNA damage after single oral exposures as high as 4 mg/kg compared to significant effects after i.p. exposure to ≥40 µg/kg [65]. The i.p. route and in vitro systems do not include possible roles of the gastrointestinal tract (GI tract) in detoxification or transfer of MCs into the vascular system. Levels of MCLR in livers 1h post-dosing were found to be 71.5% of an i.p. dose and <1% of compound given by the oral route [66]. The rates of GI tract transfer to the bloodstream by the oral route appear to be low compared to blood levels attained by the i.p. route. The types of MCLR toxicity induced by i.p. and oral administrations are similar [67,68] in the mouse and rat [69]. 

Sex-related differences in susceptibility to MCs have been reported. Significantly increased ALT and AST levels in MCLR-dosed CD-1 mice of both sexes have been documented, but females were more affected [70]. In contrast, greater hepatocellular hypertrophy and necrosis were noted in males. The studies reported here in the BALB/c mouse do not replicate these findings because ALT and AST increased to similar extents in both sexes. Significant changes in GLDH and BUN, however, occurred at mid and higher dose levels in females only. Moribundity in males was greater than females across congeners. The biological significance, if any, of these sex-related differences has not been characterized. 

MCs induced a wide range of adverse effects after exposures to 7 mg/kg for many of the congeners. This study provides a step in establishing comparative toxicity of MC congeners. We have followed this initial research by conducting multi-dose bioassays of five of the most common congeners (MCLA, MCLR, MCLY, MCRR, and MCYR) (manuscript in preparation). In the studies reported here, four of the congeners (MCRR, [Asp^3^]MCRR, [Asp^3^,Dhb^7^]MCRR, and MCWR) induced very little toxicity with a decrease in serum glucose in the male MCWR group, and an increase in liver score in the MCWR female group (the mean liver score of 3.5 was still in the acceptable range of normal based on our control mouse data in multiple studies (data not shown)). Animals dosed with either MCLF or MCLW exhibited a similar pattern of responses consisting of a trend towards higher liver weights in females and decreased glucose in males. The relative lack of toxicity of MCLF and MCLW are of note because they have been reported to be more toxic than MCLR in multiple in vitro systems including primary murine brain cells [71], primary human hepatocytes and human embryonic kidney cells (HEK293) [50], and Caco-2 cells [72]. MCs in HEK293 cell cultures transfected with recombinant human OATP1B1 and OATP1B3 differed significantly in their uptake and degree of cellular toxicity with MCLW, MCLF > MCLR > MCRR, corresponding with relative transport into hepatocytes. It has been shown that protein phosphatases PP1 and PP2A have similar inhibition by MCLW, MCLF, MCLR and MCRR in cell-free systems [50] and the resultant assumption was that substitution of hydrophobic amino acids phenylalanine (MCLF) and tryptophan (MCLW) for less-hydrophobic arginine (MCLR, MCRR) would result in increased passage through membranes [73] and higher congener concentrations interacting with phosphatases. The data presented here indicate that in vitro systems, although valuable for elucidating MC mechanisms of action, may not accurately reflect comparative in vivo MC toxicities. This inconsistency may be related to the role of gastrointestinal absorption, or other factors in MC toxicity that cannot be accounted for with in vitro systems.

This study found a similar lack of differences in toxicity between MCWR and MCRR, where the presence of tryptophan instead of arginine was not associated with significantly increased toxicity. MCYR exhibited a different pattern of toxicity compared to the other congeners evidenced by the lack of effects in liver weight or serum markers of liver toxicity (ALT, AST, and GLDH). The remaining three toxins (MCLA, MCLR, and MCLY) all induced significant toxicity in exposed animals. MCLR and MCLY have generally similar patterns of toxicity with MCLR having greater effects for weight change, liver score, ALT, AST, GLDH, BUN/creatinine, and glucose, whereas the effects on liver weight and L/BW were greater in the MCLY group. 

The yellow-tinged serum, urine, and strongly elevated serum bilirubin levels observed in MCLA are associated with jaundice due to liver or bile duct dysfunction and/or inflammation. The extent of these jaundice-associated effects was not seen within any other congeners regardless of degree of toxicity. The finding of jaundice in the sea otter poisoning event of 2007 [29] is of interest because icterus in oral mucous membranes and cartilage, and high bilirubin levels were reported following ingestion of bioaccumulated MCs in shellfish by affected animals. This took place during a period when there were MCLA-producing HABs in freshwaters that entered Monterey Bay where the incident took place. The MCs in shellfish included MCLR, MCLA, MCYR and MCRR of varying proportions in different animals. Although the studies did not discuss the possible sources of the jaundice, based upon our studies, the possibility that MCLA may have played a role in these effects should be considered. 

These studies demonstrate significant differences in the acute oral toxicity of single administrations of MC congeners at 7 mg/kg. Toxicity that included effects on ≥ seven endpoints that are indicators of toxicity occurred with four MCs (MCLA, MCLR, MCLY, and MCYR). The other congeners (MCLF, MCLW, MCRR, and MCWR) induced fewer significant signs of toxicity. The relative toxicities were not consistent with those determined by in vitro tests, emphasizing the need for in vivo approaches to evaluate the types of MC toxicity and their severities. Oral toxicity involves MCs crossing the gastrointestinal barrier, subsequent transfer to the liver via the blood, crossing hepatocyte cell membranes using Oatp carriers, and toxic reactions within the hepatocytes that include inhibition of protein phosphatases and associated cellular dysfunction, and death [1,45,50,54].

The i.p. and oral routes of administration differ in the absence and presence of MC-GI tract interactions and possible barrier functions. A comparison of the relative toxicity of other MC congeners administered by either the i.p. or oral routes may be useful for assessing the roles of the gastrointestinal tract barrier in the final extent of hepatic toxicity. These studies are currently ongoing and should increase our ability to evaluate the possible role(s) of the gastrointestinal tract on the relative toxicity of MCs. In addition to possible GI tract factors, different MC congener toxicities may involve different mechanisms of hepatic toxicity exemplified by MCLA-induced jaundice. A major data gap involves the absence of reliable data on the metabolic fate and subsequent excretion of MCs, due partly to analytical difficulties that have hindered studies designed to measure hepatic levels after exposures. Possible roles of congener-specific hepatic detoxification and excretion pathways are presently unknown. MCs are a common type of cyanobacterial toxin in U.S. freshwaters [44,74] and many basic questions about these toxins have not been studied including effects from oral sub-chronic exposures; the characterization of the toxic effects of MC congeners that have not been previously tested but are known to occur in the environment including MCAR and MCYM and various demethylated congeners, as well as adverse effects from exposures to MC mixtures.

## 4. Materials and Methods 

### 4.1. Animals

BALB/c mice (10 to 12 weeks old) with equal numbers of males and females were obtained from Charles River Laboratories (Raleigh, NC, USA). After the arrival of animals at the National Health and Environmental Effects Research Laboratory (NHEERL) animal facility, they were acclimated for at least 5 days prior to dosing. Animals were housed three per cage in polycarbonate cages on heat-treated pine shaving bedding. All cages were placed on the racks in random positions. The animal rooms had a controlled temperature range (22–26 °C) and a 12:12-h light–dark cycle. Animals were fed commercial rodent chow (Purina Prolab) and water ad libitum. Prior to the beginning of the study, the cage groups are normalized for weight, then assigned to treatment groups randomly. All studies were designed and conducted using guidelines in the U.S. Public Health Service, Policy on the Humane Care and Use of Laboratory Animals [75]. The Laboratory Animal Protocol for Research #18-09-002 was approved by the Center for Public Health and Environmental Assessment, Institutional Animal Care and Use Committee on 10 February, 2015. 

### 4.2. Compounds

MCs were reconstituted into stock solutions (Table 3) using Picopure^®^ water at a target concentration of 1 g/L. To ensure 100% reconstitution of the dry MCs, stock solutions were sonicated for 1h, followed by overnight refrigeration and an additional hour of sonication. Individual dosing solutions were prepared for each dosing group by diluting stock solutions with Picopure^®^ water. Dosing concentrations were calculated assuming a dose volume of 0.2ml, and a mouse weight equivalent to the average weight of mice in each group being dosed. For dose volumes >0.2 mL, the mice were dosed with a maximum of 0.2 mL at a one-hour interval.

The concentration of each stock solution was verified using an Agilent 6210 series time of flight mass spectrometer (MS-TOF) coupled to an Agilent 1100 series liquid chromatograph (LC). MC stock solutions were quantified against a reference material purchased from an independent vendor for each MC (Table 3) with two separate methods (i.e., external calibration curves (Figure S1) and standard addition curves (Figure S2)). All stock solutions were verified to be within 25% of the target concentrations except for MCRR (Table 3). For MCRR, the concentration in the stock solution was verified to be lower than the target, so it was assumed to be 0.85 g/L in dosing solution preparation. 

For [Asp^3^] MCRR and [Asp^3^,Dhb^7^] MCRR, purification and verification of the stock solution concentrations were completed by GreenWater Laboratories (Palatka, FL). Purification was accomplished with semi-preparative HPLC using a Phenomenex Luna C18 150 × 10 mm column and mobile phases containing acetonitrile and 5 mM ammonium bicarbonate. Quantitation was based on calibration curves comprised of certified reference MCRR, using HPLC-UV.

### 4.3. Dosing, Animal Observation and Necropsy

Animals were weighed the day prior to dosing and randomly assigned to treatment groups. Initially assigned groups were adjusted to keep the body weight variance <1 g. MCs were administered by gavage using a 20g feeding needle (Perfectum^®^, New Hyde Park, NY, USA). Animals were dosed in the morning and immediately after, placed in metabolism cages to allow urine collection for validation of techniques to analyze microcystin levels in humans [76]. The number of animals ranged from 12 to 29 per MC congener and consisted of equal numbers of males and females exposed to the MCs a single time. Two dosing regimens were used: the first was a dose response study with MCLR at 3 to 9 mg/kg that was used to determine the dose to be administered in the comparative toxicity study of the other congeners. The second study involved dosing animals with 7 mg/kg of all 10 congeners (MCLA, MCLF, MCLR, MCLW, MCLY, MCRR, [Asp^3^]MCRR, [Asp^3^,Dhb^7^]MCRR, MCWR, MCYR), based on the dose–response data obtained with MCLR. Each compound was administered to three blocks of animals received from the breeder at different time points except for the [Asp^3^]MCRR and [Asp^3^,Dhb^7^]MCRR groups that were studied in two blocks. The MC-treated animals received the toxin in 0.2 mL of Picopure^®^ water and controls received 0.2 mL of Pico-pure^®^ water alone. They were monitored for the initial 0.5h post-dosing and at hourly intervals after that for six hours. Animals were euthanized and necropsied 24h after dosing. 

We used “moribundity” rather than “lethality” as an endpoint because this allowed us to obtain maximum information from treated animals. All decisions on the moribundity classification were made by a veterinarian (DH) and there were a small number of animals that died between the time they were classified as moribund and the time they were due to be euthanized. Moribundity was defined by hunching, non-responsiveness to interaction, inappetence and/or lethargy, hypothermia, diarrhea, and/or weight loss greater than 10%, and these animals immediately were removed from their cages, euthanized, necropsied, and blood and tissues were collected for analysis as needed. The classification of moribund enabled us to obtain blood and tissue samples from animals that otherwise would have died before further data could be obtained. We find the term MD50 (50% of moribundity expressed) is a useful assessment and allows for a higher yield of data and a more humane treatment of the research animals.

Necropsies were done experimenter blind. Animals were anesthetized by CO2 inhalation, weighed, and euthanized by obtaining blood by cardiac puncture with a 25 g, 5/8 in. needle attached to a 1 mL syringe. Whole blood for clinical chemistry was transferred to a 0.5-mL serum separator tube, allowed to clot for approximately 1h, and centrifuged at 1300 × g for 2 min to separate the serum. Necropsies were performed immediately after blood collection, and before any organs were removed, or the liver weighed, a gross assessment of liver appearance was recorded that was converted to a liver score. The liver score was based on presence/severity of lesions and extent of liver surface area affected. Lesions were the visual anomalies of congestion/hemorrhage, infiltration of glycogen or lipid, or a reticulated pattern (pronounced pattern of the lobules). The scores given to individual animals ranged from 1-16, and normal was considered a score of 0–2, mild 3–5, moderate 6–8, and severe ≥9. Scoring for all animals was done by a veterinarian (DH).

### 4.4. Clinical Chemistry

All serum clinical chemistry analyses were carried out using the Randox Daytona Plus instrument (Belfast, UK). Hepatocellular injury was assessed by determining the serum activities of alanine aminotransferase (ALT), aspartate amino transferase (AST), glutamate dehydrogenase (GLDH), and bilirubin. Markers of potential renal injury included serum concentrations of blood urea nitrogen (BUN) and creatinine. Serum glucose, total protein, and albumin were also measured as markers of general toxicity. All assays were performed using reagents obtained from the instrument manufacturer.

### 4.5. Statistical Analysis

All analyses were done using SASv13.1 [77]. All variables were analyzed separately by sex. For each variable, an overall test that there were no differences in means among the treatment groups was performed. If this hypothesis was rejected (*p* < 0.05) then each treatment group was compared with the control group. This was an exploratory study, therefore no adjustments were made for multiple comparisons and *p* < 0.05 was considered statistically significant. For this reason, the pattern of significance across congeners and variables should be emphasized rather than individual p-values. The results indicated that LA was more toxic than LR in multiple outcome variables, therefore an additional post-hoc comparison was added to the analysis to test if the difference was statistically significant. 

Continuous variables were analyzed with mixed effects linear models (SAS Proc Mixed), essentially one-way ANOVAs, looking for any effect of congener. Each of the MCs were administered to different groups of animals in at least two time points, one or more weeks apart, over a period of three months. Block was included as a random effect in the model to address any block to block variability. Observations were assigned a weight of 2 for clinical chemistry values, where two (or more) samples needed to be combined to get sufficient volume for the measurement. All others were assigned a weight of 1. Levene’s test (Proc GLM) and the Shapiro–Wilk test (Proc Univariate) were used to examine homogeneity of variance and normality of the data on both the log (base 10) and linear (original) scales. If the log scale improved these properties, the variable was analyzed on the log scale; otherwise the linear scale was used. For continuous variables, the F test of treatment group effect was used to determine if there were differences among the groups, and if *p* < 0.05, pairwise t-tests relative to control were performed for each congener.

For the categorical analyses of the liver score and percent dead/moribund, there were not sufficient sample sizes to include block in the analysis. Liver score was analyzed with the mixed model ANOVA described above as well as a categorical analysis. The Cochran–Mantel–Haenszel (CMH) statistic testing for differences among group means scores was used for the overall test of treatment for liver score, and the CMH trend test was used for individual comparisons of congener with control. For percent dead/moribund, a Pearson chi-square was used as the overall test and Fisher’s Exact tests were used for the individual comparisons. The categorical analyses were conducted in Proc Freq.

## Figures and Tables

**Table 1:** MCLR Toxicity after oral administration in the BALB/c mouse^1.^

	**Number of Animals**		**Moribundity (%)**		**Weight Change (g.)**		**Liver wt (g.)**	
Dose	Male	Female	Male	Female	Male	Female	Male	Female
0	6	6	0	0	−1.47 ± 0.28	−1.72 ± 0.17	1.03 ± 0.09	0.79 ± 0.06
3	6	6	0	0	−1.82 ± 0.28	−1.73 ± 0.17	1.05 ± 0.09	0.82 ± 0.06
5	6	6	33	0	−2.62 ± 0.28^**^	−2.62 ± 0.17^***^	1.30 ± 0.09	0.82 ± 0.06
7	6	6	50	17	−2.18 ± 0.31	−2.52 ± 0.17^**^	1.31 ± 0.10	1.01 ± 0.06^**^
9	6	9	100^**^	100^***^	−1.34 ± 0.31	−0.50 ± 0.15^***^	1.69 ± 0.10^***^	1.45 ± 0.05^***^
	**Liver/Body wt**		**ALT (log_10_(IU/L))**		**AST (log_10_(IU/L))**		**GLDH (log_10_(IU/L))**	
Dose	Male	Female	Male	Female	Male	Female	Male	Female
0	5.33 ± 0.43	4.71 ± 0.28	1.89 ± 0.28	1.76±0.26	2.08 ± 0.23	2.11 ± 0.24	1.55 ± 0.20	1.30 ± 0.18
3	5.67 ± 0.43	5.09 ± 0.28	2.37 ± 0.28	2.13±0.29	2.39 ± 0.23	2.22 ± 0.26	1.76 ± 0.20	1.51 ± 0.20
5	6.91 ± 0.43^*^	5.26 ± 0.28	3.19 ± 0.28^**^	3.00±0.26^**^	2.85 ± 0.23^*^	2.82 ± 0.24^*^	2.03 ± 0.20	2.10 ± 0.18^**^
7	7.21 ± 0.47^**^	6.18 ± 0.28^***^	3.76 ± 0.31^***^	3.84±0.26^***^	3.19 ± 0.26^**^	3.55 ± 0.24^***^	2.37 ± 0.22	2.66 ± 0.18^***^
9	8.66 ± 0.47^***^	8.31 ± 0.23^***^	3.91 ± 0.35^***^	3.39±0.27^***^	3.34 ± 0.29^**^	3.20 ± 0.24^**^	2.41 ± 0.25	1.82 ± 0.18
	**Liver Score**		**BUN (mg/dl)**		**Creatinine (mg/dl)**		**BUN/Creatinine**	
Dose	Male	Female	Male	Female	Male	Female	Male	Female
0	3.00	0.67	8.59 ± 0.76	6.92 ± 0.87	0.36 ± 0.03	0.42 ± 0.03	23.78 ± 4.17	16.61 ± 2.13
3	4.00	1.00	9.96 ± 0.76	7.45 ± 0.96	0.43 ± 0.03	0.39 ± 0.03	23.48 ± 4.17	18.96 ± 2.34
5	11.00^*^	9.00^**^	9.53 ± 0.76	8.98 ± 0.87^*^	0.37 ± 0.03	0.43 ± 0.03	26.05 ± 4.17	21.12 ± 2.13
7	13.00^**^	10.50^**^	10.30 ± 0.84	11.43 ± 0.87^**^	0.36 ± 0.04	0.43 ± 0.03	28.73 ± 4.59	26.41 ± 2.13^**^
9	15.83^**^	16.0^***^	9.26 ± 0.95	9.43 ± 0.88^*^	0.32 ± 0.04	0.44 ± 0.03	38.17 ± 5.19	22.41 ± 2.15^*^
	**Albumin (g/dl)**		**Globulin (g/dl)**		**Total Protein (g/L)**		**Glucose (mg/dl)**	
Dose	Male	Female	Male	Female	Male	Female	Male	Female
0	2.96 ± 0.19	3.30 ± 0.17	1.61 ± 0.14	1.65 ± 0.09	4.72 ± 0.30	4.96 ± 0.26	176.3 ± 25.8	204.4 ± 45.5
3	3.43 ± 0.19	3.30 ± 0.19	2.07 ± 0.14^*^	1.69 ± 0.10	5.51 ± 0.30	5.00 ± 0.28	163.3 ± 25.8	115.6 ± 50.0
5	3.09 ± 0.19	3.52 ± 0.17	1.71 ± 0.14	1.78 ± 0.09	4.79 ± 0.30	5.30 ± 0.26	129.8 ± 25.8	97.1 ± 45.5
7	2.87 ± 0.20	3.28 ± 0.17	1.69 ± 0.16	1.61 ± 0.09	4.57 ± 0.33	4.89 ± 0.26	75.4 ± 28.4	77.1 ± 45.5
9	2.16 ± 0.23^*^	2.56 ± 0.17^**^	1.25 ± 0.18	1.29 ± 0.09^*^	3.42 ± 0.37^*^	3.85 ± 0.26^**^	132.6 ± 32.1	215.1 ± 46.0
	**Total Bilirubin (mg/dl)**							
Dose	Male	Female						
0	0.17 ± 0.07	0.21 ± 0.08						
3	0.20 ± 0.06	0.18 ± 0.09						
5	0.37 ± 0.08	0.22 ± 0.08						
7	0.38 ± 0.07	0.33 ± 0.08						
9	0.25 ± 0.08	0.37 ± 0.08						

^1^ All significance tests of difference from control. Mortality statistical significance by Fisher’s exact test; liver score by Cochran–Mantel–Haenszel chi-square; all other endpoints by ANOVA/t-test. ^*^ *p* < 0.05 for differences from controls. ^**^ *p* < 0.01 for differences from controls. ^***^ *p* < 0.001 for differences from controls.

**Table 2:** Comparison of MCLR and MCLA levels of toxicity - controls for reference^1^.

Variable	Control Male	Female	MCLA Male	MCLR Male	MCLA Female	MCLR Female
Number of animals	24	24	8	14	8	15
Moribundity (%)	0	0	100 **	29	63 **	7
Weight Change (g.)	−1.68 ± 0.18	−1.43 ±0.09	−1.30 ± 0.30 ***	−2.66 ± 0.22	−2.02 ± 0.16 **	−2.66 ± 0.11
Liver Wt (g.)	1.06 ± 0.05	0.82 ± 0.03	1.89 ± 0.10***	1.26 ± 0.07	1.26 ± 0.05***	0.94 ± 0.04
Liver/Body wt ratio	5.06 ± 0.23	4.75 ± 0.18	9.09 ± 0.39***	6.58 ± 0.29	7.56 ± 0.23 ***	5.77 ± 0.20
Liver score (avg.)	2.21 ± 0.44	0.17 ± 0.16	16 ± 0 *	12.10 ± 0.97	13.75 ± 1.40 **	9.20 ± 0.82
ALT (log10 (IU/L))	1.94 ± 0.16	1.99 ± 0.16	4.37 ± 0.35 *	3.43 ± 0.19	4.05 ± 0.27	3.50 ± 0.19
AST (log10 (IU/L))	2.17 ± 0.11	2.27 ± 0.11	3.99 ± 0.27***	2.97 ± 0.14	4.38 ± 0.24***	3.17 ± 0.14
GLDH (log10 (IU/L))	1.40 ± 0.12	1.43 ± 0.13	2.71 ± 0.23*	2.25 ± 0.14	1.95 ± 0.23	2.38 ± 0.15
BUN (mg/dL)	8.58 ± 0.66	8.37 ± 0.50	12.90 ± 1.37 *	9.90 ± 0.77	11.93 ± 0.91	10.56 ± 0.62
Creatinine (mg/dL)	0.45 ± 0.03	0.50 ± 0.02	0.51 ± 0.04	0.45 ± 0.03	0.37 ± 0.03**	0.48 ± 0.03
BUN/Creatinine ratio	19.39 ± 1.66	16.67 ± 0.91	24.88 ± 2.53	22.24 ± 1.78	29.24 ± 1.64**	22.23 ± 1.12
Albumin (g/dL)	3.43 ± 0.15	3.74 ± 0.19	2.72 ± 0.23***	3.45 ± 0.16	4.39 ± 0.28	3.69 ± 0.21
Globulin (g/dL)	1.90 ± 0.15	1.80 ± 0.17	1.33 ± 0.20***	1.92 ± 0.16	1.67 ± 0.19	1.65 ± 0.17
Total Protein (g/dL)	5.37 ± 0.21	5.49 ± 0.21	4.09 ± 0.33***	5.36 ± 0.23	6.11 ± 0.33	5.31 ± 0.24
Glucose (mg/dL)	215.3 ± 11.6	217.3 ± 11.6	95.8 ± 25.2	103.6 ± 14.7	82.2 ± 17.9	97.0 ± 13.4
Total bilirubin (mg/dL)	0.23 ± 0.09	0.24 ± 0.15	1.29 ± 0.11***	0.38 ± 0.09	4.47 ± 0.23***	0.27 ± 0.17

^1^ Control values for reference. Moribundity statistical significance by Fisher’s exact test; liver score by Cochran–Mantel–Haenszel chi-square; all other endpoints by ANOVA/t-test. Differences of MCLA compared to MCL.R * *p* < 0.05; ** *p* < 0.01; and *** *p* < 0.001.

**Table 3:** Microcystin stock solutions quantified with an external calibration curve and standard addition.

[M-H]^-^		Dosing Solution Vendor	External Calibration Quantification (g/L)	Standard Addition Quantification (g/L)	Reference Material Vendor
**MCLF**	984.5083	Enzo Lot #30325	0.94 ± 0.12	1.3	Sigma Aldrich Lot #BCBS3614V
**MCLY**	1000.5032	Enzo Lot #30326	0.91 ± 0.05	1.1	Abraxis Lot#L3032060917
**MCRR**	1036.558	Beagle Lot #MCRR-2004	0.74 ± 0.06	0.75	NRC Lot #200702071111
**MCLR**	993.541	Enzo Lot #30362	1.0 ± 0.11	0.88	NRC Lot #200701311388
**MCWR**	1066.5355	Enzo Lot #L30471	1.1 ± 0.03	1.2	Abraxis Lot # 300652
**MCLA**	908.4772	Beagle Lot #MCLA-2009	1.1 ± 0.11	1	Abraxis Lot #B1071017
**MCYR**	1043.5052	Enzo Lot #L30470	1.3 ± 0.21	1	Sigma Aldrich Lot #BCBR8280V
**MCLW**	1023.5192	Enzo Lot #30392	1.1 ± 0.29	1	Sigma Aldrich Lot #BCBS5567V

## Supplementary Materials

The following are available online at https://www.mdpi.com/2072-6651/12/6/403/s1, Table S1: Gross toxicity and clinical data for microcystin congeners; Figure S1: Calibration curves of the congeners using external quantification; Figure S2: Calibrations curves of the congeners using standard addition method.
