# Supplementary material for: The Comparative Toxicity of 10 Microcystin Congeners Administered Orally to Mice: Clinical Effects and Organ Toxicity
Source: Toxins (Basel). 2020 Jun 18;12(6):403. doi: 10.3390/toxins12060403 (PMC7354475; doi:10.3390/toxins12060403)
Supplement: Supplementary file 1 [file toxins-12-00403-s001.pdf]

Supplementary Materials: The Comparative Toxicity of Ten Microcystin Congeners Administered Orally to Mice: Clinical Effects and Organ Toxicity

Neil Chernoff, Donna Hill, Johnsie Lang, Judy Schmid, Thao Le, Amy Farthing and Hwa Huang

Table S1. Gross toxicity and clinical data for microcystin congeners[a].

Variable	Control Male	Control Female	MCLA Male	MCLA Female
N	30	30	8	8
Moribundity (%)	0	0	100.0 ***	62.5 **
Wt Change %	−7.36 ± 0.62	−8.18 ± 0.58	−6.08 ± 1.18	−10.98 ± 0.99 **
Weight change (g)	−1.72 ± 0.16	−1.55 ± 0.12	−1.32 ± 0.29	−2.07 ± 0.19 **
Liver Wt (g)	1.09 ± 0.05	0.82 ± 0.03	1.90 ± 0.09 ***	1.26 ± 0.04 ***
Liver/body wt	5.03 ± 0.20	4.73 ± 0.16	9.08 ± 0.36 ***	7.54 ± 0.22 ***
ALT (IU/L)	1.89 ± 0.14	1.98 ± 0.14	4.36 ± 0.32 ***	4.05 ± 0.25 ***
AST (IU/L)	2.15 ± 0.09	2.29 ± 0.09	3.98 ± 0.25 ***	4.41 ± 0.22 ***
GLDH (IU/L)	1.37 ± 0.10	1.45 ± 0.11	2.69 ± 0.21 ***	1.97 ± 0.21 *
Liver score	2.60 ± 0.42	0.40 ± 0.24	16.00 ± 0.00 ***	13.75 ± 1.40 ***
BUN (mg/dL)	8.64 ± 0.57	8.72 ± 0.42	12.94 ± 1.29 **	11.93 ± 0.85 **
Creatinine (mg/dL)	0.46 ± 0.02	0.51 ± 0.02	0.52 ± 0.04	0.37 ± 0.03 ***
BUN/Creatinine	19.18 ± 1.39	16.80 ± 0.76	24.68 ± 2.37 *	29.24 ± 1.54 ***
Albumin (g/dL)	3.43 ± 0.15	3.74 ± 0.19	2.72 ± 0.23 ***	4.39 ± 0.28 *
Globulin (g/dL)	1.90 ± 0.15	1.80 ± 0.17	1.33 ± 0.20 ***	1.67 ± 0.19
Total protein (g/dL)	5.42 ± 0.19	5.56 ± 0.18	4.13 ± 0.31 ***	6.13 ± 0.31
Glucose (mg/dL)	212.78 ± 10.24	219.13 ± 10.47	95.04 ± 24.62 ***	83.66 ± 18.30 ***
Tot. bilirubin (mg/dL)	0.21 ± 0.08	0.22 ± 0.13	1.27 ± 0.10 ***	4.47 ± 0.21 ***
**Variable**	**MCLF Male**	**MCLF Female**	**MCLR Male**	**MCLR Female**
N	9	9	14	15
Moribundity (%)	0	0	28.6 *	6.7
Wt Change %	−8.57 ± 1.01	−9.99 ± 0.91	−12.11 ± 0.85 ***	−14.20 ± 0.73 ***
Weight change (g)	−1.97 ± 0.25	−1.92 ± 0.18 *	−2.68 ± 0.21 ***	−2.71 ± 0.15 ***
Liver Wt (g)	1.10 ± 0.08	0.91 ± 0.04 *	1.27 ± 0.07 *	0.94 ± 0.04 ***
Liver/body wt	5.26 ± 0.31	5.30 ± 0.21 **	6.57 ± 0.27 ***	5.75 ± 0.18 ***
ALT (IU/L)	2.01 ± 0.20	2.01 ± 0.22	3.41 ± 0.17 ***	3.50 ± 0.18 ***
AST (IU/L)	2.24 ± 0.15	2.32 ± 0.17	2.97 ± 0.13 ***	3.18 ± 0.13 ***
GLDH (IU/L)	1.57 ± 0.14	1.52 ± 0.17	2.23 ± 0.12 ***	2.39 ± 0.13 ***
Liver score	1.89 ± 1.14	2.78 ± 1.18 *	12.07 ± 0.97 ***	9.20 ± 0.82 ***
BUN (mg/dL)	9.51 ± 0.83	9.07 ± 0.75	9.94 ± 0.72	10.56 ± 0.58 *
Creatinine (mg/dL)	0.49 ± 0.03	0.49 ± 0.03	0.46 ± 0.03	0.49 ± 0.03
BUN/Creatinine	19.53 ± 1.72	17.17 ± 1.36	22.04 ± 1.57 *	22.23 ± 1.05 ***
Albumin (g/dL)	3.51 ± 0.18	3.75 ± 0.25	3.45 ± 0.16	3.69 ± 0.21
Globulin (g/dL)	2.00 ± 0.17	1.73 ± 0.18	1.92 ± 0.16	1.65 ± 0.17
Total protein (g/dL)	5.60 ± 0.23	5.58 ± 0.28	5.40 ± 0.21	5.35 ± 0.23
Glucose (mg/dL)	182.13 ± 15.55	188.60 ± 16.65	102.22 ± 14.13 ***	97.64 ± 13.34 ***
Tot. bilirubin (mg/dL)	0.25 ± 0.09	0.57 ± 0.19	0.37 ± 0.08 ***	0.27 ± 0.16
**Variable**	**MCLW Male**	**MCLW Female**	**MCLY Male**	**MCLY Female**
N	9	9	8	8
Moribundity (%)	0	0	25	0
Wt Change %	−9.13 ± 1.01	−8.83 ± 0.91	−9.08 ± 1.05	−13.68 ± 0.95 ***
Weight change (g)	−2.14 ± 0.25	−1.67 ± 0.18	−2.11 ± 0.26	−2.56 ± 0.18 ***
Liver Wt (g)	1.16 ± 0.08	0.92 ± 0.04 **	1.48 ± 0.08 ***	0.96 ± 0.04 ***
Liver/body wt	5.49 ± 0.31	5.37 ± 0.21 ***	6.84 ± 0.33 ***	5.93 ± 0.21 ***
ALT (IU/L)	2.28 ± 0.20	2.19 ± 0.22	2.63 ± 0.23 **	2.56 ± 0.23 *
AST (IU/L)	2.48 ± 0.15 *	2.47 ± 0.17	2.57 ± 0.18 *	2.78 ± 0.18 *
GLDH (IU/L)	1.72 ± 0.14 **	1.75 ± 0.17	2.15 ± 0.16 ***	1.90 ± 0.17 *
Liver score	2.33 ± 1.21	2.22 ± 0.60 **	7.75 ± 2.28 *	5.50 ± 1.59 **
BUN (mg/dL)	9.45 ± 0.83	9.41 ± 0.75	9.69 ± 0.95	8.89 ± 0.80

Creatinine (mg/dL)	0.47 ± 0.03	0.51 ± 0.03	0.45 ± 0.03	0.45 ± 0.03 *
BUN/Creatinine	20.25 ± 1.72	17.25 ± 1.36	21.70 ± 1.88	19.12 ± 1.44
Albumin (g/dL)	3.48 ± 0.18	3.86 ± 0.25	3.36 ± 0.19	3.51 ± 0.26
Globulin (g/dL)	2.03 ± 0.17	1.77 ± 0.18	1.85 ± 0.18	1.63 ± 0.18
Total protein (g/dL)	5.59 ± 0.23	5.74 ± 0.28	5.29 ± 0.25	5.23 ± 0.29
Glucose (mg/dL)	168.63 ± 15.55 **	208.43 ± 16.65	113.79 ± 17.95 ***	134.68 ± 17.39 ***
Tot. bilirubin (mg/dL)	0.27 ± 0.09	0.20 ± 0.21	0.26 ± 0.09	0.67 ± 0.20 *

**Variable**	**MCRR Male**	**MCRR Female**	**[Asp^{3}]MCRR Male**	**[Asp^{3}]MCRR Female**
N	9	8	6	6
Moribundity (%)	0	0	0	0
Wt Change %	−8.18 ± 1.01	−8.34 ± 0.95	−8.68 ± 1.35	−6.87 ± 1.22
Weight change (g)	−1.84 ± 0.25	−1.57 ± 0.18	−2.15 ± 0.34	−1.25 ± 0.24
Liver Wt (g)	1.06 ± 0.08	0.85 ± 0.04	1.14 ± 0.11	0.83 ± 0.05
Liver/body wt	5.20 ± 0.31	4.94 ± 0.21	5.05 ± 0.42	4.73 ± 0.27
ALT (IU/L)	2.10 ± 0.20	1.94 ± 0.23	1.79 ± 0.27	1.88 ± 0.30
AST (IU/L)	2.27 ± 0.15	2.20 ± 0.18	2.17 ± 0.20	2.28 ± 0.21
GLDH (IU/L)	1.53 ± 0.14	1.52 ± 0.17	1.38 ± 0.18	1.28 ± 0.22
Liver score	3.00 ± 0.58	1.25 ± 0.84	3.67 ± 0.84	0.33 ± 0.33
BUN (mg/dL)	8.51 ± 0.83	8.92 ± 0.80	6.82 ± 1.11	7.99 ± 0.92
Creatinine (mg/dL)	0.47 ± 0.03	0.46 ± 0.03	0.44 ± 0.03	0.50 ± 0.04
BUN/Creatinine	18.42 ± 1.72	18.04 ± 1.44	16.20 ± 2.16	14.24 ± 1.66
Albumin (g/dL)	3.32 ± 0.17	3.61 ± 0.26	n.o.	n.o.
Globulin (g/dL)	1.85 ± 0.17	1.64 ± 0.18	n.o.	n.o.
Total protein (g/dL)	5.24 ± 0.23	5.33 ± 0.29	5.46 ± 0.29	5.49 ± 0.38
Glucose (mg/dL)	208.80 ± 15.48	200.73 ± 17.39	197.83 ± 20.88	186.86 ± 23.71
Tot. bilirubin (mg/dL)	0.22 ± 0.09	0.31 ± 0.21	0.23 ± 0.10	0.17 ± 0.26

**Variable**	**[Asp^{3},Dhb^{7}]MCRR Male**	**[Asp^{3},Dhb^{7}]MCRR Female**	**MCWR Male**	**MCWR Female**
N	5	6	6	6
Moribundity (%)	0	0	0	0
Wt Change %	−7.19 ± 1.43	−6.52 ± 1.22	−8.76 ± 1.20	−8.65 ± 1.07
Weight change (g)	−1.89 ± 0.35	−1.17 ± 0.24	−1.98 ± 0.29	−1.66 ± 0.21
Liver Wt (g)	1.25 ± 0.11	0.88 ± 0.05	1.09 ± 0.10	0.81 ± 0.05
Liver/body wt	5.02 ± 0.45	5.04 ± 0.27	5.22 ± 0.37	4.63 ± 0.23
ALT (IU/L)	1.67 ± 0.29	1.93 ± 0.30	1.64 ± 0.24	1.67 ± 0.27
AST (IU/L)	1.99 ± 0.21	2.27 ± 0.21	2.01 ± 0.18	1.99 ± 0.20
GLDH (IU/L)	1.28 ± 0.19	1.30 ± 0.22	1.11 ± 0.16	1.04 + 0.20 *
Liver score	1.80 ± 1.11	0.83 ± 0.54	3.33 ± 0.71	3.50 ± 0.72 **
BUN (mg/dL)	8.30 ± 1.17	8.60 ± 0.92	9.47 ± 0.96	7.48 ± 0.92
Creatinine (mg/dL)	0.46 ± 0.04	0.49 ± 0.04	0.49 ± 0.03	0.49 ± 0.03
BUN/Creatinine	18.27 ± 2.24	15.77 ± 1.66	17.38 ± 1.90	15.42 ± 1.66
Albumin (g/dL)	n.o.	n.o.	3.60 ± 0.19	3.57 ± 0.30
Globulin (g/dL)	n.o.	n.o.	1.93 ± 0.18	1.71 ± 0.19
Total protein (g/dL)	5.52 ± 0.30	5.43 ± 0.38	5.63 ± 0.25	5.27 ± 0.33
Glucose (mg/dL)	213.42 ± 21.98	199.34 ± 22.38	159.11 ± 18.27 **	187.76 ± 19.81
Tot. bilirubin (mg/dL)	0.21 ± 0.10	0.17 ± 0.26	0.25 ± 0.09	0.08 ± 0.23

**Variable**	**MCYR Male**	**MCYR Female**
N	9	9
Moribundity (%)	11.1	0
Wt Change %	−11.58 ± 1.01 ***	−9.80 ± 0.91
Weight change (g)	−2.79 ± 0.25 ***	−1.83 ± 0.18
Liver Wt (g)	1.16 ± 0.08	0.85 ± 0.04
Liver/body wt	5.45 ± 0.33	5.09 ± 0.21 *
ALT (IU/L)	2.29 ± 0.21	2.30 ± 0.22
AST (IU/L)	2.27 ± 0.16	2.59 ± 0.17
GLDH (IU/L)	1.65 ± 0.14 *	1.53 ± 0.17
Liver score	7.89 ± 1.32 **	3.89 ± 0.99 **
BUN (mg/dL)	11.68 ± 0.86 ***	9.39 ± 0.75
Creatinine (mg/dL)	0.50 ± 0.03 *	0.51 ± 0.03

BUN/Creatinine	23.26 ± 1.76 **	18.51 ± 1.36
Albumin (g/dL)	3.86 ± 0.18 ***	3.61 ± 0.26
Globulin (g/dL)	2.05 ± 0.17	1.67 ± 0.18
Total protein (g/dL)	5.99 ± 0.24 **	5.31 ± 0.28
Glucose (mg/dL)	145.02 ± 16.15 ***	172.12 ± 16.66 **
Tot. bilirubin (mg/dL)	0.31 ± 0.09 *	0.17 ± 0.19

[a] * $p < 0.05$, ** $p < 0.01$, *** $p < 0.001$.

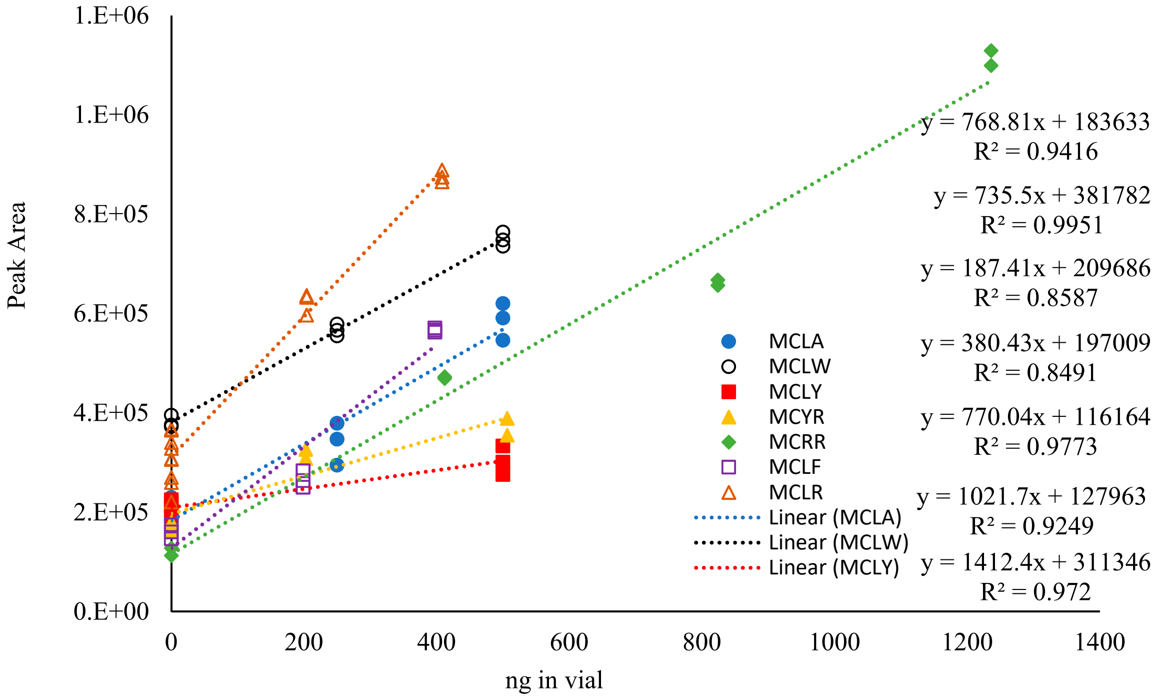

Figure S1. Calibration curves for microcystin congeners created using a standard addition quantification method.

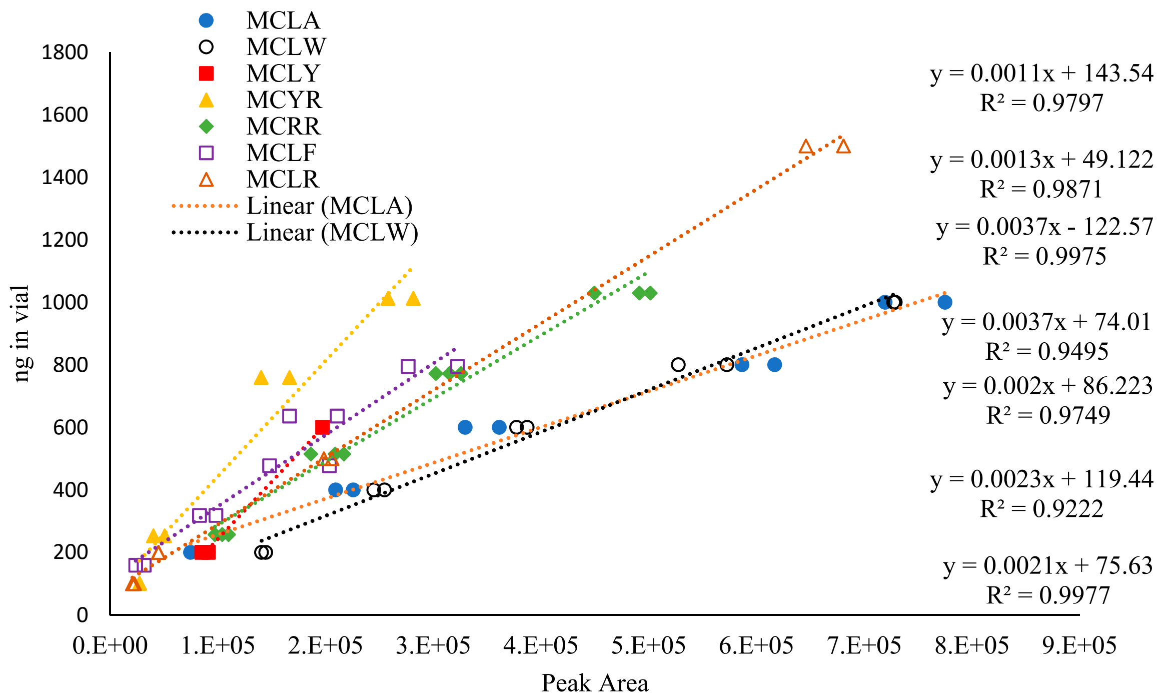

Figure S2. Calibration curves for microcystin congeners created using an external quantification method.
